# Construction of an organic cage-based porous ionic liquid using an aminal tying strategy†

**DOI:** 10.1039/d5me00004a

**Published:** 2025-04-02

**Authors:** Aiting Kai, Austin Mroz, Kim E. Jelfs, Andrew I. Cooper, Marc A. Little, Rebecca L. Greenaway

**Affiliations:** a Department of Chemistry and Materials Innovation Factory, University of Liverpool 51 Oxford Street Liverpool L7 3NY UK; b Department of Chemistry, Molecular Sciences Research Hub, Imperial College London 82 Wood Lane London W12 0BZ UK r.greenaway@imperial.ac.uk; c I-X Centre for AI in Science, Imperial College London White City Campus W12 0BZ London UK; d Institute of Chemical Sciences, Heriot-Watt University Edinburgh EH14 4AS UK

## Abstract

An aminal tying method was applied to post-synthetically modify a flexible organic cage, **RCC1**, to construct a porous ionic liquid (PIL). The resulting PIL, [**RCC1**-IM][NTf_2_]_6_, displayed melting behaviour below 100 °C, a transition to a glass phase on melt-quenching, CO_2_ uptake, and its permanent porosity was confirmed using molecular dynamic simulations.

## Abstract 2

An organic cage-based porous ionic liquid (PIL) was constructed by functionalising a reduced cage core with ionic liquid functionality. The resulting PIL displayed melting behaviour, a glass phase, enhanced CO_2_ uptake and permanent porosity.

## Graphical abstract

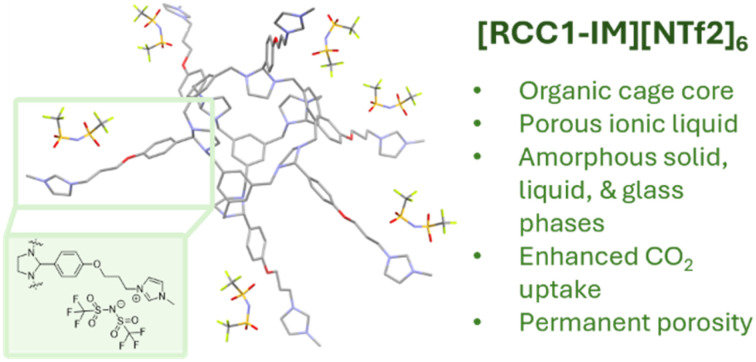

**Design, System, Application**

Porous liquids (PLs) realize permanent microporosity in the liquid state and are well-suited to a range of applications. Porous ionic liquids (PILs) represent a sub-class of PLs, which are porogens incorporated with ionic liquids as functional groups or as solvents. The available porosity of a PIL depends on the concentration of pores within the system. Neat PILs, which consist of a liquid porogen without any solvents present to effectively dilute the pores, offer the potential of accessing the highest theoretical pore volumes in a liquid. However, the challenge lies in the difficulty of designing large, shape-persistent pore carriers that melt at reasonable temperatures. Here, we construct a neat PIL *via* post-synthetic modification of a low-molecular weight porous organic cage; specifically, an imine cage was reduced and aminal-tied with aldehydes to reintroduce some structural rigidity into the cage. An ionic liquid moiety was pre-incorporated into the aldehydes, resulting in a porous molecular structure that melts at the desired temperature. The resulting bulk material was an amorphous solid but displayed melting behaviour over 55–90 °C; this successfully demonstrated the inheritance of ionic liquid properties from the aldehydes to the cage itself. Porosity of the PIL was confirmed through both experiments and computation.

## Introduction

Porous liquids (PLs) are defined as liquids that contain permanent microporosity – they can be neat systems where the porosity originates in the liquid porogen itself (type I), solutions of porogens dissolved in a cavity-excluded solvent (type II), or dispersions of porogens dispersed in a cavity-excluded solvent (type III).^1^ Porous ionic liquids (PILs) are a sub-class of PLs which incorporate ionic liquid (IL) functionality, whether that is on the pore carrier itself or as the solvent,^2^ and offer certain advantages, not least the negligible vapor pressures of most systems.

The first type I PIL was reported by Nitschke and co-workers, where ionic liquid functionality was incorporated onto the periphery of a metal–organic cage (MOC).^3^ Dai and co-workers reported a supramolecular complexation strategy between an anionic porous organic cage (POC) and different crown-ethers to form type II PLs with similar properties to type I PILs.^4^ Moving towards other molecular organic species, other examples of macrocyclic ILs include cyclodextrins^5^ and pillar[5]arenes,^6^ which were both post-synthetically modified with ionic liquid functionality, with the latter reported to exhibit significant CO_2_ uptake (5.52 mol mol^−1^ at 1 bar). A similar approach has been applied to metal–organic framework nanocrystals by surface modification with ionic liquids.^7^ Type II and type III PILs have also been reported. For example, MOCs have been dissolved in a handful of ILs.^8,9^ Similarly, MOFs,^10,11^ zeolitic-imidazolate frameworks (ZIFs),^12,13^ zeolites,^14^ hollow carbon nanospheres,^15^ POC microparticles,^16^ and covalent organic framework (COF) particles,^17^ have all been dispersed in ionic liquids using different strategies. However, compared to type II and III PILs, type I PILs could offer higher pore volumes due to the lack of a fluidising solvent, alongside improved stability and processability. However, type I PILs arguably remain the most challenging to realise experimentally, with it often proving difficult to render large molecular pore carriers meltable. Ideally, such PILs should be liquid at or near room temperature while maintaining the shape persistence of the pore carrier and avoiding interpenetration of the fluidising periphery functionalisation into the cavities to ensure permanent porosity is present in the liquid state.

Here, we report the synthesis of an organic cage-derived neat porous ionic liquid, [**RCC1**-IM][NTf_2_]_6_ (where IM = 3-(3-(4-formylphenoxy)propyl)-1-methyl-imidazolium, and NTf_2_ = bis(trifluoromethanesulfonyl)imide), formed using a post-functionalisation strategy. Previously, Liu *et al.* reported the reduction of imine-derived POCs into flexible amine cages, which could subsequently be aminal ‘tied’ with small aldehydes to re-introduce shape-persistence.^18^ These tied-reduced organic cages exhibit increased stability due to the removal of the reversible nature of the imine bond, an added advantage in targeting neat type I porous liquids. In addition, the reduced organic cages exhibit melting transitions and, as such, could also be processed into melt-quenched glass phases, which demonstrated enhanced gas uptake over the pre-processed amorphous material.^19^ This reduction-aminal formation approach was adopted here to construct an organic cage-based ionic liquid using a larger ionic-liquid functionalised aldehyde. The resulting material exhibited both a melting point below 100 °C and a glass transition on melt-quenching, with the amorphous solid, liquid, and melt-quenched glass phases all exhibiting comparable CO_2_ uptakes, and molecular dynamic (MD) simulations confirming the size-exclusivity of the selected ionic liquid anion from the organic cage cavity, and therefore, it's permanent porosity.

## Results and discussion

**Fig. 1:**
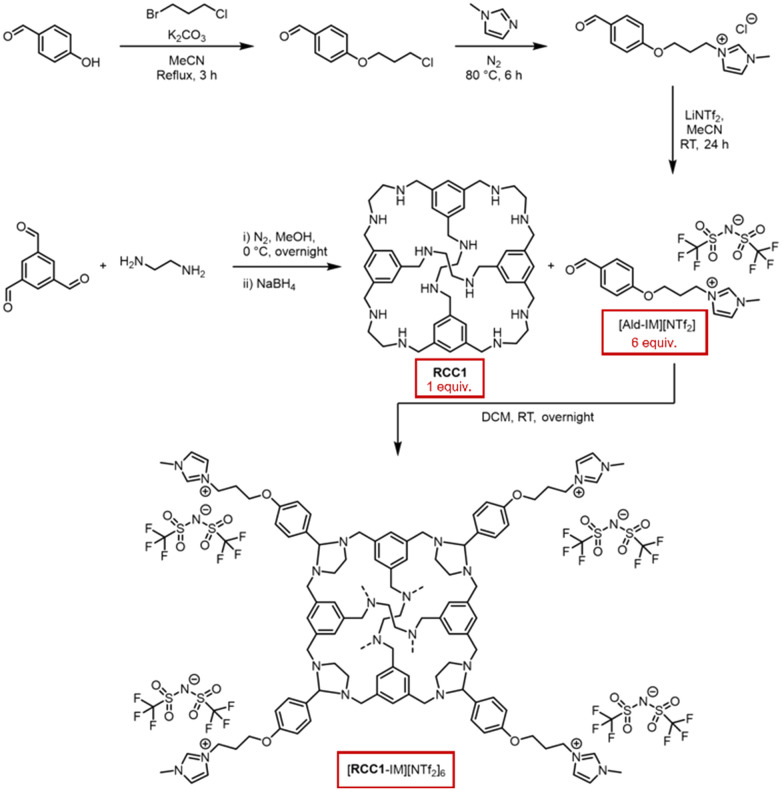
Synthesis of ionic liquid functionalised aldehyde [Ald-IM][NTf_2_], reduced organic cage **RCC1**, and formation of an ionic liquid-functionalised cage [**RCC1**-IM][NTf_2_]_6_ – [**RCC1**-IM][NTf_2_]_6_ includes six aminal-tied ionic liquid functional groups but only four are shown for clarity, with the remaining two linking together the connected diamines *via* the dashed lines.

First, a functionalised aldehyde containing a bulky imidazolium ionic liquid, denoted [Ald-IM][NTf_2_], was designed and synthesised based on known literature methods (Fig. 1).^20^ This ionic liquid derived aldehyde was initially synthesised with chloride as the counterion, but subsequently exchanged for the larger and bulkier NTf_2_ counterions – the rationale for this was two-fold. First, the behaviour of ionic compounds is typically dominated by the electrostatic forces that hold the cations and anions together, which obey Coulomb's inverse-square law:
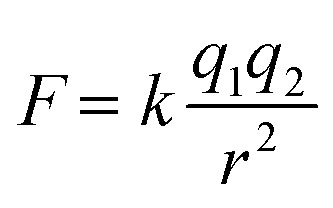
where *F* is the force, *q* is the charge of the ions, *k* is a constant, and *r* represents the distance between the cation and anion. As the electrostatic force is inversely proportional to *r*^2^, if *r* is larger, the corresponding electrostatic force will vastly decrease. Therefore, the use of bulky cations and anions will generally reduce the electrostatic interactions between the ionic species, often resulting in lower melting points.^21^ Second, we previously found this anion to be cavity-excluded in a type III based porous liquid consisting of porous organic cage microparticles, and therefore hypothesised that it would also be cavity-excluded from the targeted system here.

This aldehyde was subsequently reacted with **RCC1***via* aminal formation to form an ionic liquid-functionalised cage [**RCC1**-IM][NTf_2_]_6_. Unlike other structurally analogous reduced cages, such as **RCC3** formed using the highly pre-configured and rigid *trans*-1,2-cyclohexanediamine,^18^**RCC1** is synthesised using flexible ethylenediamine, which enables the cage vertices in **RCC1** to invert. The increased flexibility of **RCC1** enables the amine cage to react with larger aldehyde derivatives that are then subsequently arranged on the periphery of the cage. Characterisation by ^1^H NMR spectroscopy (Fig. S8†), matrix-assisted laser desorption/ionisation time-of-flight (MALDI-TOF) mass spectrometry (Fig. S11†), and elemental analysis, confirmed the formation of the six-fold tied [**RCC1**-IM][NTf_2_]_6_ (Table S1†). In particular, MALDI-TOF confirmed the predominant formation of the six-fold tied species with NTf_2_ counterions – a mass ion corresponding to the [M-NTf_2_]^+^ was observed (calc. 3577.89; found 3578.08), alongside the mass ion of the NTf_2_ counterions in negative ionisation mode (calc. 279.92; found 279.89). In addition, elemental analysis confirmed that a high-purity sample was obtained.

**Fig. 2:**
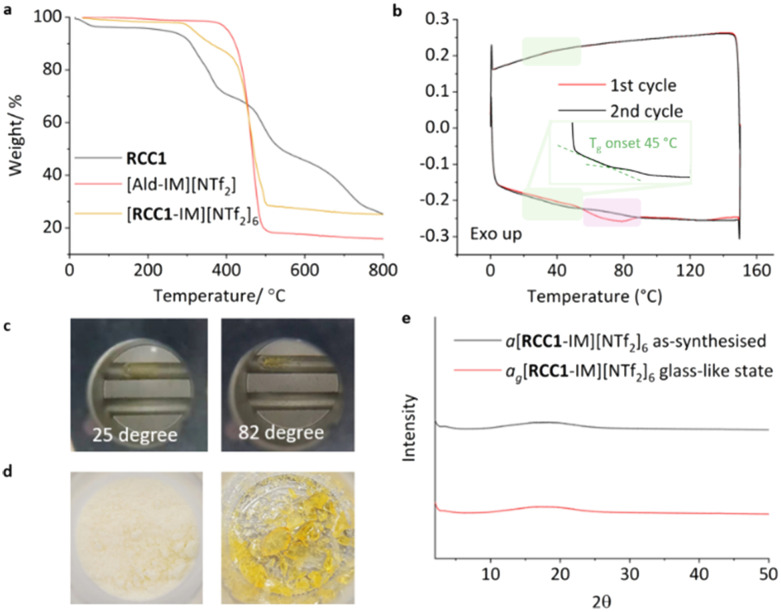
(a) TGA curves of **RCC1** (black line), [Ald-IM][NTf_2_] (red line) and [**RCC1**-IM][NTf_2_]_6_ (yellow line); (b) DSC trace of [**RCC1**-IM][NTf_2_]_6_ subjected to a heat-cool-heat cycle to 150 °C: initial upscan shown as a solid red line, with downscan and second upscan as a black line, with broad melting range highlighted in pink and a reversible glass transition highlighted in green with an enhanced inset; (c) melting behaviour of [**RCC1**-IM][NTf_2_]_6_ observed using melting point apparatus; (d) optical images of *a*[**RCC1**-IM][NTf_2_]_6_ (left) and *a*_g_[**RCC1**-IM][NTf_2_]_6_ (right); (e) PXRD patterns of as-synthesised *a*[**RCC1**-IM][NTf_2_]_6_ (top, black) and *a*_g_[**RCC1**-IM][NTf_2_]_6_ (bottom, red).

The as-synthesised [**RCC1**-IM][NTf_2_]_6_ was isolated as an off-white powder, and found to be amorphous by powder X-ray diffraction (PXRD) (Fig. 2d). Thermogravimetric analysis (TGA) was performed to evaluate its thermal stability compared to both **RCC1** and [Ald-IM][NTf_2_], with a featureless trace identified up to the decomposition temperatures (*T*_d_) in excess of 300 °C (Fig. 2a). Following this, a staged temperature loss is observed between 300–400 °C prior to complete decomposition – on comparison to the traces for **RCC1** and [Ald-IM][NTf_2_], this can be potentially attributed to the aminals being cleaved leading to the cage core beginning to thermally decompose, followed by the ionic liquid components fully decomposing. Differential scanning calorimetry (DSC) was subsequently carried out, heating the samples to below their *T*_d_ before cooling to room temperature, and then reheating once more. [Ald-IM][NTf_2_] exhibited a relatively sharp endotherm, which correlated with a melting point of 58 °C (Fig. S7†). [**RCC1**-IM][NTf_2_]_6_, on the other hand, exhibited a broad endotherm (Fig. 2b) over the 55–90 °C temperature range, with a subtle but reversible glass transition observed at ∼40 °C. The broad endotherm of [**RCC1**-IM][NTf_2_]_6_ was unambiguously identified as a melting transition, *T*_m_, by monitoring the melting range using melting point apparatus (Fig. 2c). As observed previously, we believe the broad melting range is due to the amorphous nature of the as-synthesised material.^22^ While [**RCC1**-IM][NTf_2_]_6_ was not a liquid at room temperature, the system can be classed as an ionic liquid, according to the definition of ionic liquids being organic salts that have a melting point below 100 °C.^23^

In relation to the formed glassy-material recovered at room temperature after melt-quenching, as shown in the optical image (Fig. 2c), PXRD confirmed that the material remained amorphous (Fig. 2d). Subsequent analysis by mass spectrometry also confirmed the presence of intact [**RCC1**-IM][NTf_2_]_6_ (Fig. S11†), ruling out the possibility of bond breaking without mass loss over the DSC temperature range, and confirming the formation of a molecular amorphous glass. The glassy material could also be processed back into its amorphous powder by soaking in dichloromethane and drying under a vacuum, demonstrating a solution processability advantage over framework-based glasses.^19,24^ In line with previous terminology, the amorphous solid and amorphous glass formed here are referred to as *a*[**RCC1**-IM][NTf_2_]_6_ and *a*_g_[**RCC1**-IM][NTf_2_]_6_, respectively, and the liquid is referred to as *l*[**RCC1**-IM][NTf_2_]_6_.

**Fig. 3:**
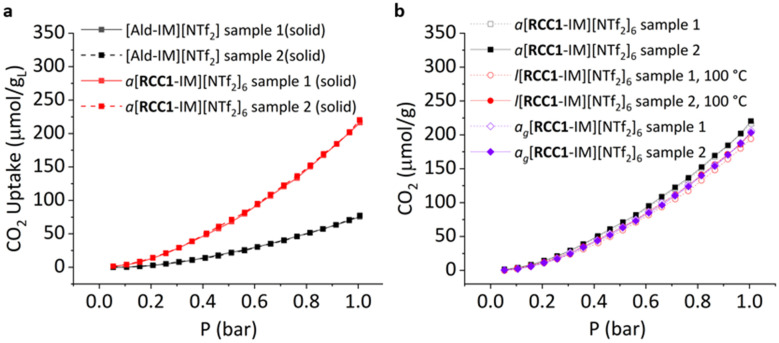
(a) CO_2_ adsorption isotherms for [Ald-IM][NTf_2_] (black) and *a*[**RCC1**-IM][NTf_2_]_6_ (red) at 298 K; (b) CO_2_ adsorption isotherms for the different phases of [**RCC1**-IM][NTf_2_]_6_: solid *a*[**RCC1**-IM][NTf_2_]_6_ at 298 K (black squares), liquid *l*[**RCC1**-IM][NTf_2_]_6_ at 373 K (red circles), and solid *a*_g_[**RCC1**-IM][NTf_2_]_6_ at 298 K (purple diamonds).

Next, sorption studies were carried out to investigate the porosity of the material in its different phases. First, the CO_2_ uptakes of both the [Ald-IM][NTf_2_] and *a*[**RCC1**-IM][NTf_2_]_6_ were measured for comparison (298 K, 1 bar), and to see the effect on uptake on incorporation of the **RCC1** core (Fig. 3a). The quantity of CO_2_ adsorbed by *a*[**RCC1**-IM][NTf_2_]_6_ was found to be threefold higher than just the bulky imidazolium ionic liquid [Ald-IM][NTf_2_] (218 ± 2.8 μmol g^−1^*vs.* 76 ± 1.3 μmol g^−1^, respectively). This enhancement in the solid state can be attributed to the incorporation of permanent intrinsic porosity from the cage core alongside additional extrinsic porosity afforded by poor-packing of the highly decorated POCs. In addition, when taking into account the molecular weights of the two materials, the CO_2_ solubility in *a*[**RCC1**-IM][NTf_2_]_6_ far exceeds that of [Ald-IM][NTf_2_] (0.78 *vs.* 0.04 mol_CO_2__mol_IL_^−1^, respectively) with the latter being similar to analogous ionic liquids such as [BMIM][NTf_2_] (75 ± 2.8 μmol g^−1^, 0.03 mol_CO_2__mol_IL_^−1^).^16^ Subsequently, the CO_2_ uptake was measured at 373 K (100 °C), enabling the uptake of the organic-cage derived ionic liquid *l*[**RCC1**-IM][NTf_2_]_6_ to be assessed in its melted state (Fig. 3b). While the volume of the sample visibly reduced on melting, and stirring of the thin layer of clear yellow viscous liquid was not possible, the measured uptake of *l*[**RCC1**-IM][NTf_2_]_6_ (198 ± 6.58 μmol g^−1^; 0.71 mol_CO_2__mol_IL_^−1^) was similar to that of the solid *a*[**RCC1**-IM][NTf_2_]_6_ at 298 K. Again, this level of enhancement (∼2.6 fold) in a PL is not unusual or unexpected, with gas uptakes in previously reported POC-based type II porous liquids (which inherently have lower pore volumes compared to type systems due to the presence of solvent) known to demonstrate up to 8-fold increases.^25^ Finally, the CO_2_ uptake after cooling back to 298 K was repeated to study the uptake of the *a*_g_[**RCC1**-IM][NTf_2_]_6_ material, which was also similar to both the amorphous and liquid phases (204 ± 1.68 μmol g^−1^; 0.73 mol_CO_2__mol_IL_^−1^). Measurements were also repeated over two samples to ensure reproducibility. For context, the CO_2_ uptake in this organic cage-based ionic liquid is comparable to that of our previously reported 20 wt% type III porous liquid of **CC3**-*R*/**CC3**-*S* in [BPy][NTf_2_] at room temperature (209.8 ± 6.7 μmol g^−1^),^16^ and the CO_2_ uptake in *a*_g_[**RCC1**-IM][NTf_2_]_6_ is on the same order of magnitude as our previously reported melt-quenched glass formed from **RCC3**,^22^ albeit slightly lower, which is likely due to the increased number of secondary amines in the latter.

Finally, to confirm the existence of permanent porosity in [**RCC1**-IM][NTf_2_]_6_ and the size-exclusion of the NTf_2_ counterions, MD simulations were performed at the experimental conditions for the liquid material (1 bar, 363 K). A simulation box was constructed featuring 48 cage molecules and 288 anions, retaining the required 1 : 6 cage : anion ratio (Fig. S12†). The starting lattice was equilibrated to a reasonable density of 1.342 ± 0.005 g cm^−3^ (Fig. S13, Table S2†), comparable to the experimental densities of analogous ionic liquid systems. The [**RCC1**-IM][NTf_2_]_6_ system was simulated for 10 ns. The liquid configuration was analysed by comparing the radial distribution function of [**RCC1**-IM][NTf_2_]_6_ with an analogous ionic liquid, [BMIM][NTf_2_] (Fig. S14†). We observe peaks in the same positions as those reported in the literature for the analogous ionic liquid; this suggests that the simulation is performing as expected.^26^

**Fig. 4:**
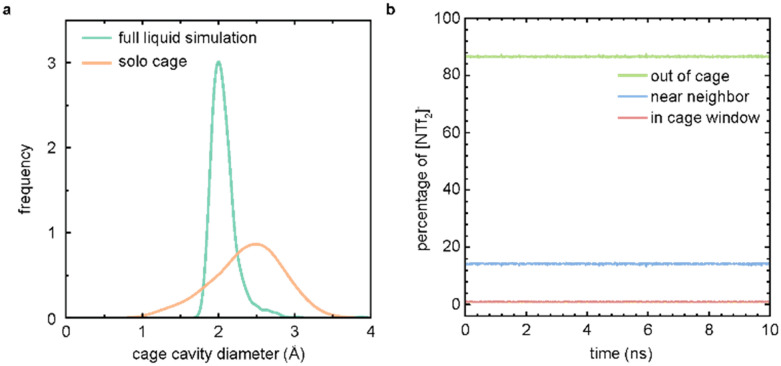
(a) Cage cavity diameter distribution over the course of the full liquid simulation (teal) and a simulation featuring one cage molecule simulated in gas (orange); (b) percentage of the NTf_2_ counterions in cage windows (red), near neighbour (blue), and in the bulk solvent (green) over the course of the 10 ns simulation.

To examine the porosity exhibited by [**RCC1**-IM][NTf_2_]_6_, we compared the distribution of the cage cavity diameters over the full MD simulation and a simulation featuring only a single cage molecule in isolation (Fig. 4a). The cavity diameter distribution for the full liquid simulation was generated using Zeo++,^27,28^ and presents a histogram of the largest cavity diameter at each sampled timestep of the simulation. The distribution for the solo cage simulation is obtained from an MD simulation of a single cage molecule in gas; the histogram of the largest cavity diameter at each timestep is calculated using *pyWindow*.^29^ The distribution of cage cavity diameters over the course of the liquid simulation falls within the distribution for the cage cavity diameters from a simulation of a single cage molecule. This overlap further demonstrates that the observed void space in the full liquid simulation is not solely due to the transient void space inherent to the IL structure of the system, and is additionally supported by void space analyses of similar, neat imidazolium-based ILs.^30^ This supports the hypothesis that the observed experimental gas uptake is due to the porosity of the [**RCC1**-IM][NTf_2_]_6_ system. The narrower distribution of the cage cavity diameters from the full liquid simulation is caused by the pressure of the surrounding counterions, which impede the dynamics of the cage molecule compared to a single molecule in the gas phase.

To further verify that the measured gas uptake is indicative of a PIL, we tracked the anions over the course of the simulation (Fig. 4b). Here, we observe that at any given time, ∼86% of the NTf_2_ counterions are near a cage molecule (within 20 Å of the centre of a cage), ∼13% reside in the bulk liquid phase (>20 Å away from a cage centre), and ∼0.09% are located at a cage window (∼5.5 Å away from the cage centre). These cutoffs are determined by the geometry of the cage molecule. Importantly, no NTf_2_ counterions were observed to enter the cage cavity during the simulations. These results support the initial hypothesis that the NTf_2_ counterions are too large to diffuse into the [**RCC1**-IM]^6+^ cavity, and the [**RCC1**-IM][NTf_2_]_6_ system is a PIL. Further, these results are similar to a previously published study where the location of the solvent hexachloropropene was tracked over the course of a porous liquid simulation featuring scrambled **CC3**^**3**^**13**^**3**^ as the porous motif.^31^ Here, ∼80% of solvent molecules reside in the bulk, ∼16% reside near a neighbour, and 3% are located in a cage window. Full simulation analysis details for the [**RCC1**-IM][NTf_2_]_6_ system are presented in Table S3.†

## Conclusions

Post-synthetic functionalisation of a reduced amine cage with an ionic liquid functionalised aldehyde *via* aminal formation led to the successful formation of an organic cage decorated with ionic liquid functionality, [**RCC1**-IM][NTf_2_]_6_. This cage was initially isolated as an amorphous material, but exhibited melting behaviour over 55–90 °C, and on subsequent quenching exhibited a glass transition and could be processed into a transparent glassy state. Furthermore, the material could be re-processed into its original non-glassy amorphous state. The as-synthesised amorphous solid [**RCC1**-IM][NTf_2_]_6_ showed a CO_2_ uptake three times that of solid [Ald-IM][NTf_2_] (218 ± 2.8 μmol g^−1^*vs.* 76 ± 1.3 μmol g^−1^), with both the liquid and glass exhibiting similar uptakes. The porosity of the system was further supported by molecular dynamics simulations which demonstrated that the observed experimental gas uptake is due to the porosity of the [**RCC1**-IM][NTf_2_]_6_ system, as opposed to the transient void space inherent to the IL structure of the system. While the presented PIL shows reduced CO_2_ uptake compared to some of the previously reported PILs discussed in the introduction, arguably the versatility of this material in relation to its processability and accessible phases (amorphous powder, liquid, or glass), which all exhibit similar gas uptake capability, is one of the main advantages of this cage-based PIL. In addition, it does not incorporate any metals and it does not require the use of an additional solvent to induce fluidity.

## Data availability

All experimental details and the data supporting this article have been included as part of the ESI.† Full details of the computational workflow for structure generation and simulation box construction of the porous ionic liquid are included in the corresponding GitHub repository – https://github.com/austin-mroz/RCC1-IM-NTf2.git.

## Conflicts of interest

There are no conflicts to declare.

## Supplementary Material

ME-010-D5ME00004A-s001
