# Supplementary material for: Construction of an organic cage-based porous ionic liquid using an aminal tying strategy
Source: Mol Syst Des Eng. 2025 Apr 2;10(6):459–63. doi: 10.1039/d5me00004a (PMC11979736; doi:10.1039/d5me00004a)
Supplement: ME-010-D5ME00004A-s001 [file ME-010-D5ME00004A-s001.pdf]

Supplementary Information (SI) for Molecular Systems Design & Engineering.
This journal is © The Royal Society of Chemistry 2025

Supplementary information

Construction of an organic cage-based porous ionic liquid using an aminal tying strategy

Aiting Kai,[a] Austin Mroz,[b,c] Kim E. Jelfs,[b] Andrew I. Cooper,[a] Marc. A. Little[a,d] and Rebecca L. Greenaway[a,b*]

[a]Department of Chemistry and Materials Innovation Factory, University of Liverpool, 51 Oxford Street, Liverpool, L7 3NY, UK.

[b]Department of Chemistry, Molecular Sciences Research Hub, Imperial College London, 82 Wood Lane, London, W12 0BZ, UK.

[c]I-X Centre for AI in Science, Imperial College London, White City Campus, W12 0BZ, London, UK

[d]Institute of Chemical Sciences, Heriot-Watt University, Edinburgh, EH14 4AS, UK.

Email: r.greenaway@imperial.ac.uk

1. General synthetic and analytical methods

Materials: 1,3,5-Triformylbenzene (TFB) was purchased from Manchester Organics (UK). Other chemicals were purchased from Fluorochem UK, TCI UK or Sigma-Aldrich. Solvents were reagent or HPLC grade purchased from Fischer Scientific. All materials were used as received unless stated otherwise.

Synthesis: All reactions were stirred magnetically using Teflon-coated stirrer bars. Where heating was required, the reactions were warmed using a stirrer hotplate with heating blocks, with the stated temperature being measured externally to the reaction flask with an attached probe. Removal of solvents was done using a rotary evaporator.

NMR: ^{1}H NMR, ^{13}C NMR and ^{19}F NMR spectra were recorded in deuterated methanol, DMSO or chloroform at 400 MHz using a Bruker Avance 400 NMR spectrometer. Chemical shifts are reported in ppm (δ) with reference to internal residual protonated species of the deuterated solvent. NMR data are presented as follows: chemical shift, integration, peak multiplicity (s = singlet, d = doublet, t = triplet, q = quartet, m = multiplet, br = broad) and coupling constants (*J* / Hz).

MALDI-TOF MS: Mass spectroscopy was measured on a BRUKER AutoFlex spectrometer. The data was collected by flexControl software and exported from the flexAnalysis software. DCTB was used as the matrix and CsI_3 was used to calibrate the instrument each time before measurements. Solutions of matrix and sample (10 mg/mL in methanol) were mixed in a ratio of 10:1 before being deposited onto a steel plate with a micropipette. Samples on the steel plate were left to evaporate to complete dryness before loaded to the spectrometer for test.

TGA: Thermogravimetric analysis was performed on a Q5000IR with an automated vertical overhead thermobalance. Samples were heated in an aluminium pan at a ramp rate of 10 °C/min up to 800 °C under a dry nitrogen flow.

DSC: Differential scanning calorimetry analysis was performed on a TA instruments Discovery DSC. Samples were weighed in Tzero aluminium hermetic pans and sealed with Tzero hermetic lid using the blue set pin hole. Samples were measured under a dry nitrogen flow, with a heating rate of 10 °C/min and a cooling rate of 5 °C/min, respectively.

Melting observation: Melting behaviour was observed on a Stuart digital melting point apparatus SMP 10. Samples were first loaded into the capillary glass tubes (length = 100 mm; inner diameter = 1.3 mm; wall thickness = 0.3 mm). The sample tube was then inserted into the heating block, and the apparatus heated until plateau which was set at 25 °C. Afterwards the temperature was ramped at a rate of 2 °C/min and the samples observed through a magnifying lens.

Gas sorption analysis: Gas uptakes were measured on a Quantachrome Nova 4200e. Generally, 100 mg sample was loaded into a 9 mm sample cell with a large bulb (P/N: 74064). The sample was degassed under vacuum with stirring overnight at room temperature and backfilled with helium before being removed from the degassing station. A filler rod (P/N: 74105-L) was used with the sample cell during sorption measurements at room temperature and the adsorption settings were as follows: 20 pressure points from 0.05 to 1.0 bar in 0.05 increments; pressure tolerance = 0.05 mmHg; equilibration time = 1800 seconds; equilibration timeout = 5400 seconds.

PXRD: Powder X-ray diffraction patterns were collected in transmission mode on samples held on a thin Mylar film in aluminium well plates on a Panalytical Empyrean diffractometer equipped with a high-throughput screening (HTS) XYZ stage, X-ray focusing mirror, and PIXcel detector, using Cu-Kα (λ = 1.541 Å) radiation. PXRD patterns were recorded at room temperature, and diffraction patterns were measured over the range of 2−50°, in 0.013° steps, for 30-60 minutes.

2. Synthesis and Characterisation

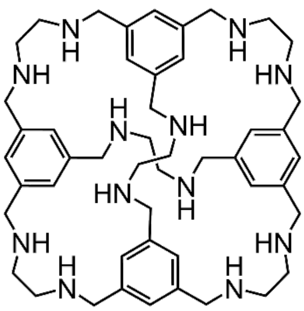

RCC1 was synthesised according to the literature with minor modifications.[1] 1,3,5-Triformylbenzene (TFB, 1.875 g, 0.0116 mol) was first dissolved in methanol (575 mL) in a 2 L round-bottomed flask cooled in an ice bath. Ethylenediamine (1.04 g, 0.0173 mol) was dissolved in methanol (425 mL) and added in batches over 1 hour to the TFB solution under a nitrogen atmosphere. The reaction mixture was then stirred overnight before confirming complete consumption of the TFB by ^{1}H NMR spectroscopic analysis. On completion, sodium borohydride (1.53 g, 0.0403 mol) was added, and the reaction mixture stirred for a further 12 hours. Water (5 mL) was then added and the reaction stirred for a further 12 hours before the solvent was removed under vacuum. The resulting colourless solid was then extracted with dichloromethane (DCM, 2 × 100 mL) and the remaining colourless solid removed by filtration. The solvent was then removed with a rotary evaporator (water bath at 30 °C) and **RCC1** was obtained as an off-white solid (1.47 g, 62%) which was used without further purification.

^{1}H NMR (400 MHz, CD_3OD) δ_H 7.10 (12H, s, H_A), 3.65 (24H, s, H_B), 2.60 (24H, s, H_C) (**Figure S1**); **^{13}C NMR** (101 MHz, CD_3OD) δ_C 141.09, 128.37, 54.09 (**Figure S2**).

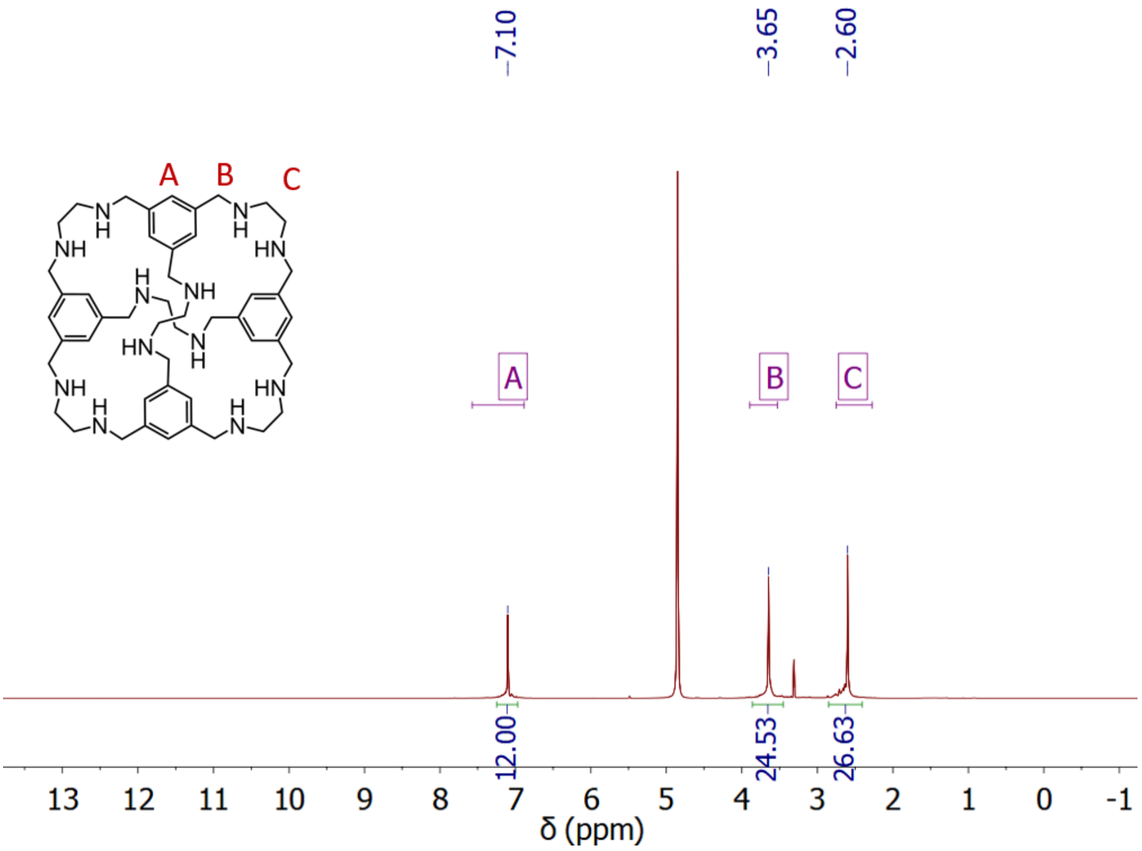

Figure S1: ^{1}H NMR (400 MHz, CD_3OD) spectra of **RCC1**.

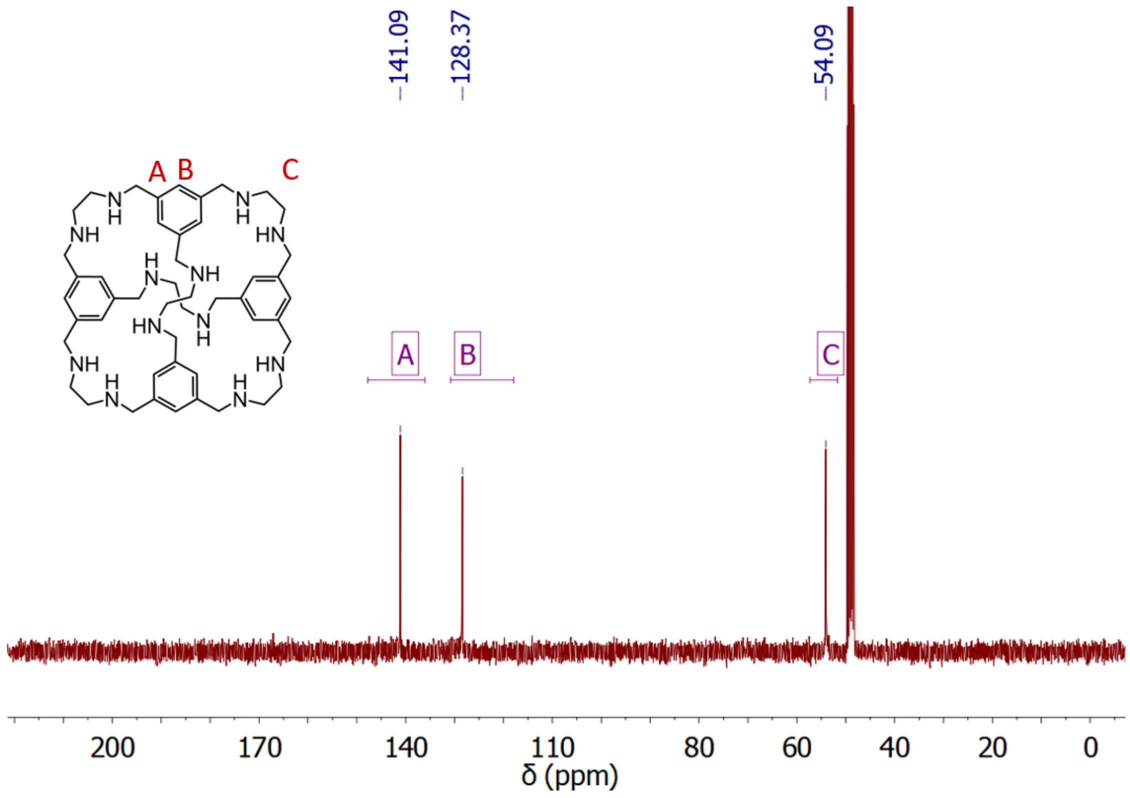

Figure S2: ^{13}C NMR (101 MHz, CD_3OD) spectra of **RCC1**.

[Ald-IM][NTf$_2$] was synthesised according to the literature with minor modifications.[2]

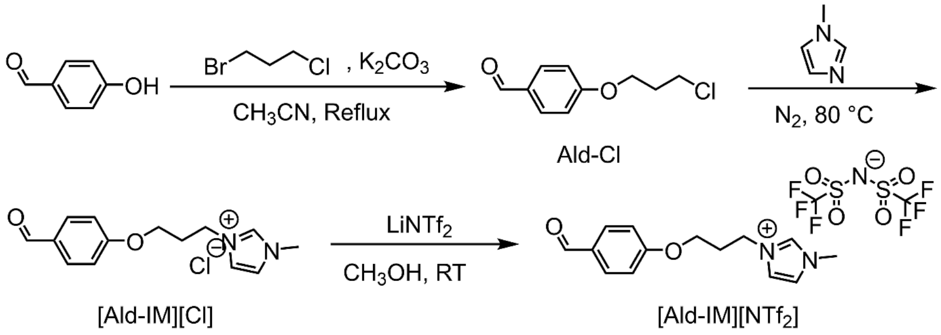

Figure S3: Synthesis route of ionic liquid functionalised aldehyde [Ald-IM][NTf$_2$].

4-(3-chloropropoxy)benzaldehyde (Ald-Cl): A mixture of *p*-hydroxybenzaldehyde (2.44 g, 10 mmol), 1-bromo-3-chloropropane (4.723 g, 30 mmol) and K_2CO_3 (5.528 g, 40 mmol) in acetonitrile (150 mL) was heated at reflux for 3 hours, during which the reaction was monitored by TLC. On completion, the acetonitrile was removed by evaporation, and the residue dispersed in DCM and filtered. The organic layer was then evaporated and purified by column chromatography (silica gel, 100% DCM) to obtain the targeted product Ald-Cl as a light-yellow oil (3.65 g, 92%).

^{1}H NMR (400 MHz, $CDCl_3$) δ_H 9.89 (1H, s, CHO), 7.84 (2H, m, ArH), 7.01 (2H, m, ArH), 4.21 (2H, t, *J* = 6 Hz, OCH_2), 3.76 (2H, t, *J* = 6 Hz, CH_2), 2.28 (2H, q, *J* = 6 Hz, CH_2); **^{13}C NMR** (101 MHz, $CDCl_3$) δ_C 190.91, 163.84, 132.14, 130.25, 130.25, 114.89, 64.74, 41.33, 32.11.

[Ald-IM][Cl]: A mixture of Ald-Cl (1.986 g, 10 mmol) and 1-methylimidazole (1.231 g, 15 mmol) was heated at 80 °C for 6 h to give a thick viscous liquid. The viscous liquid was then washed with diethyl ether (3 × 30 mL) to obtain the crude product [Ald-IM][Cl] (2.02 g, 72%) which was used without further purification.

^{1}H NMR (400 MHz, CD_3OD) δ_H 9.85 (1H, s, CHO), 9.02 (1H, s, C=NH), 7.87 (2H, m, ArH), 7.65 (2H, m, ArH), 7.07 (2H, m, ArH), 4.48 (2H, t, *J* = 7.2 Hz, OCH_2), 4.20 (2H, t, *J* = 6 Hz, CH_2), 3.93 (3H, s, CH_3), 2.43 (2H, m, CH_2); **^{13}C NMR** (101 MHz, $CDCl_3$) δ_C 192.78, 165.02, 138.19, 133.11, 131.73, 125.06, 123.92, 115.93, 66.20, 48.22, 36.50.

[Ald-IM][NTf$_2$]: [Ald-IM][Cl] (1.00 g, 3.56 mmol) was dissolved in acetonitrile (50 mL) and lithium bis(trifluoromethanesulfonyl)imide ($LiNTf_2$, 1.148 g, 4.00 mmol) was added. The mixture was then stirred at room temperature for 24 hours before the solution was diluted with DCM (100 mL), washed with water, and dried ($MgSO_4$). The mixture was filtered and the filtrate concentrated to yield [Ald-IM][NTf$_2$] as an off-white waxy solid (1.53 g, 82%) which was used without further purification.

^{1}H NMR (400 MHz, CD_3OD) δ_H 9.84 (1H, s, CHO), 7.87 (2H, m, ArH), 7.62 (2H, dd, *J* = 36, 2 Hz, IM-H), 7.07 (2H, m, ArH), 4.46 (2H, t, *J* = 7.2 Hz, CH_2), 4.19 (2H, t, *J* = 5.6 Hz, CH_2), 3.01 (3H, s, CH_3), 2.42 (2H, m, CH_2) (**Figure S4**); **^{13}C NMR** (101 MHz, CD_3OD) δ_C 192.82, 165.01, 133.12, 131.73, 125.02, 123.86, 122.80, 115.91, 66.12, 36.43, 30.51 (**Figure S5**); **^{19}F NMR** (376 MHz, CD_3OD) δ_F 80.70 (**Figure S6**). **Elemental analysis (%):** C, 36.86; H, 3.22; N, 8.05 (Calculated: C, 36.57; H, 3.26; N, 8.00).

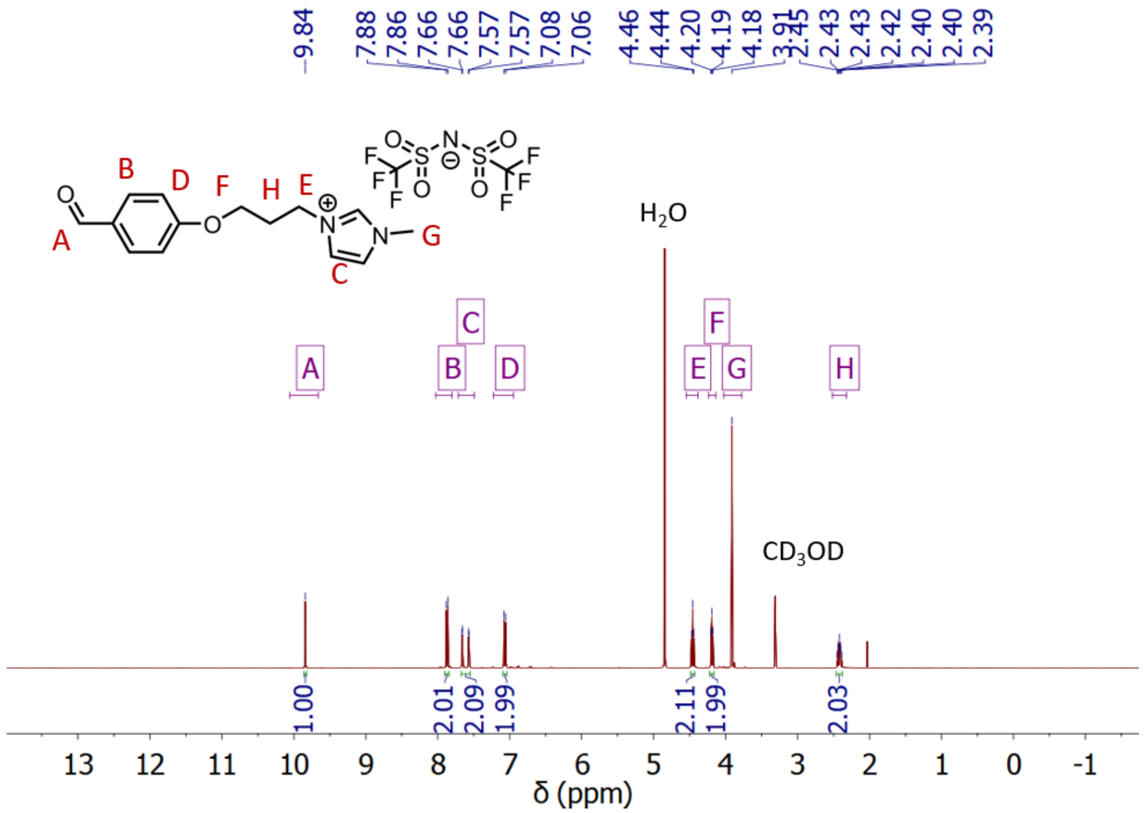

Figure S4: ^{1}H NMR (400 MHz, CD_3OD) spectra of [Ald-IM][NTf_2].

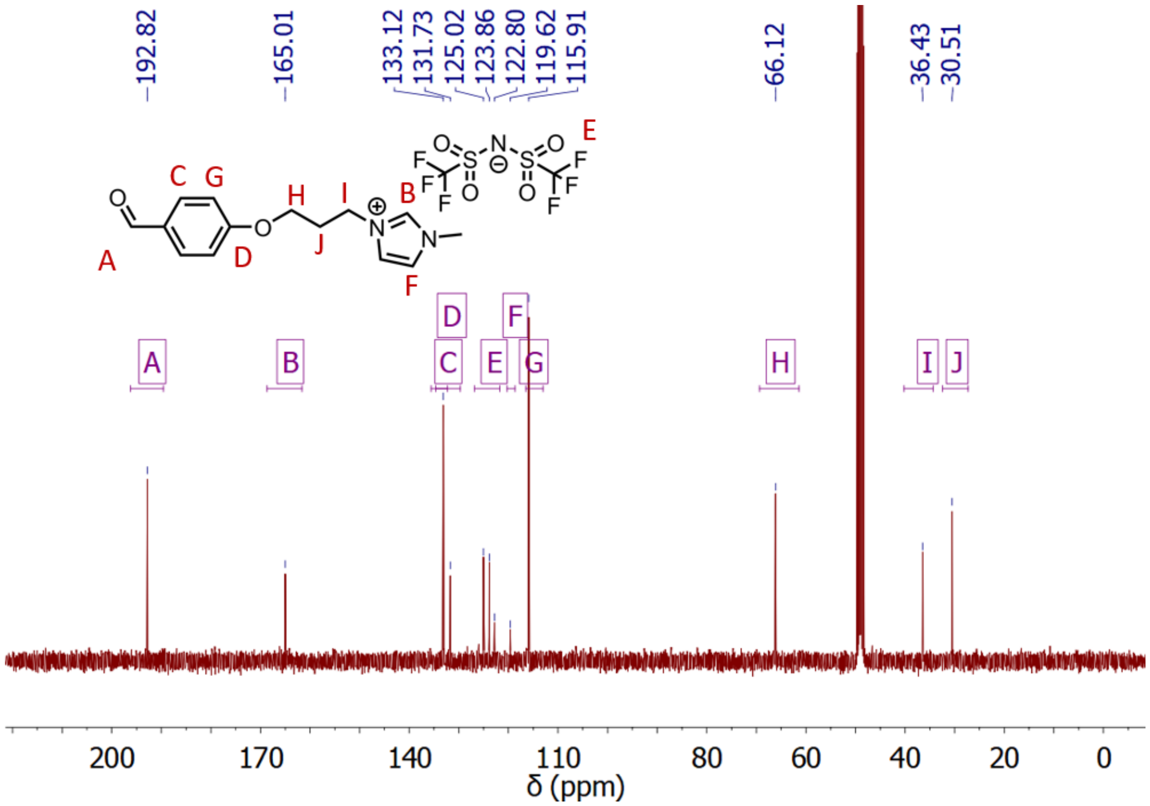

Figure S5: ^{13}C NMR (101 MHz, CD_3OD) spectra of [Ald-IM][NTf_2].

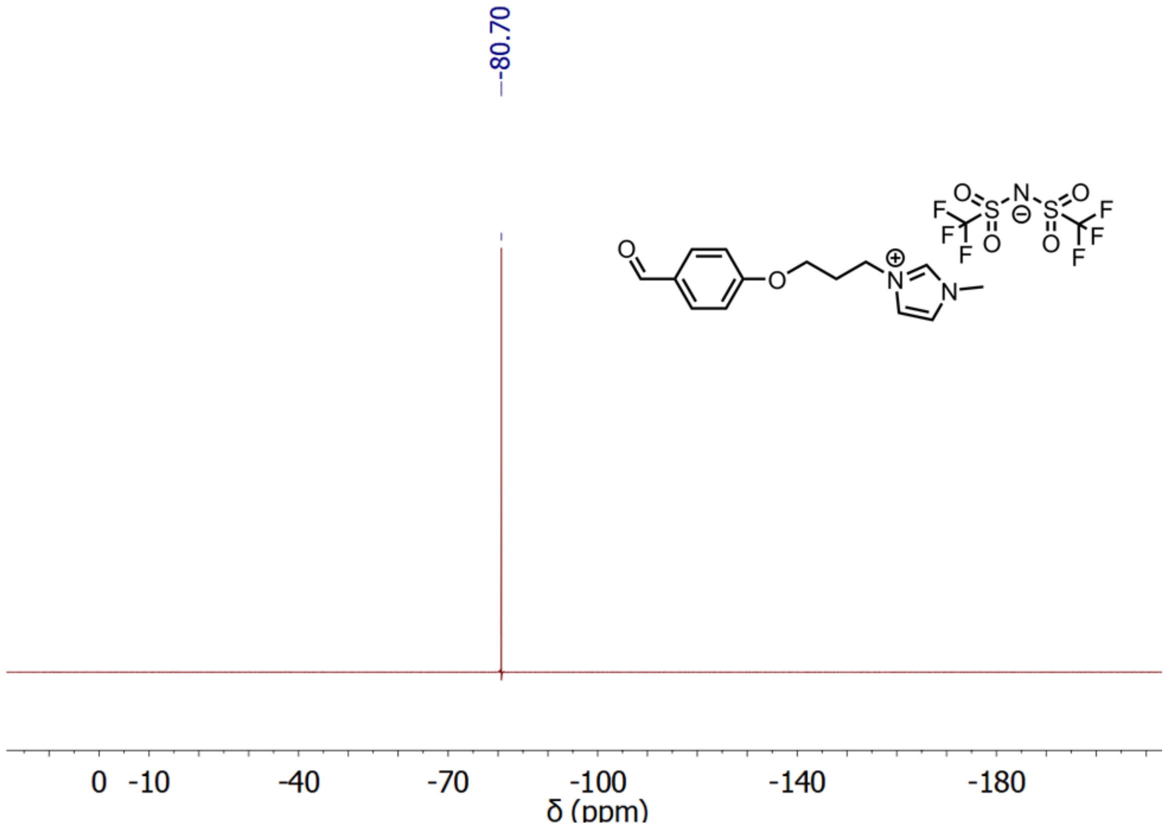

Figure S6: ^{19}F NMR (376 MHz, CD_3OD) spectra of [Ald-IM][NTf_2].

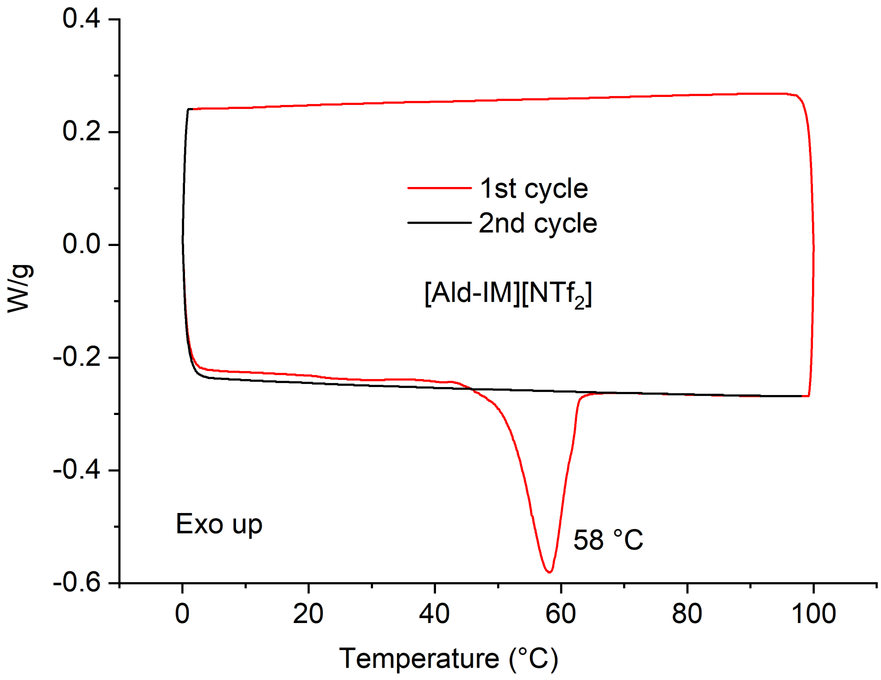

Figure S7: DSC trace of [Ald-IM][NTf_2].

[RCC1-IM][NTf$_2$]$_6$: General method of tying **RCC1** with [Ald-IM][NTf$_2$] via aminal formation was as follows. **RCC1** (30 mg, 0.037 mmol, 1 equiv.) was dissolved in DCM (2 mL) to which was added a solution of [Ald-IM][NTf$_2$] (175 mg, 0.333 mmol, 9 equiv.) in DCM (2 mL). The solution typically became opaque after a few minutes and was kept stirring overnight. The reaction mixture was then left to stand until phase separation occurred, where the lower layer of oily phase was the aminal-tied product. The upper DCM layer was decanted, and the lower product was washed with DCM several times to afford the product [**RCC1**-IM][NTf$_2$]$_6$ which was characterised and studied without further purification.

^{1}H NMR (400 MHz, DMSO-*d*$_6$) - due to the flexible nature and ionic character of the cage, broad peaks were observed (**Figure S8**). δ_H 9.26 (6H, br, H$_A$), 7.91-7.28 (24H, br m, H$_B$/H$_C$), 7.20-6.38 (24H, br m, H$_D$/H$_E$), 4.36 (12H, br, H$_F$), 4.02 (12H, br, H$_G$), 3.84 (24H, br, H$_H$), 3.58 (6H, br, H$_I$), 3.20-2.85 (12H, br m, H$_J$), 2.27 (24H, br, H$_K$); **^{13}C NMR** (101 MHz, DMSO-*d*$_6$) δ_C 137.27, 124.76, 124.04, 122.90, 121.56, 118.36, 115.16, 64.91, 46.80, 36.18, 29.64 – low intensity peaks but key signals from both **RCC1** and [Ald-IM][NTf$_2$] present (**Figure S9**); **^{19}F NMR** (376 MHz, CD$_3$OD) δ_F 78.69 (**Figure S10**); **MALDI-TOF MS (RP) m/z:** accurate mass calculated for $C_{142}H_{160}F_{30}N_{29}O_{26}S_{10}^+$ ([**RCC1**-IM]$^+$[NTf$_2$]$_5$) 3577.89, found [M-NTf$_2$]$^+$ 3578.08; **MALDI-TOF MS (RN) m/z:** accurate mass calculated for [NTf$_2$] $C_2F_6NO_4S_2^-$ 279.92, found [NTf$_2$]$^-$ 279.89; Comparison of MALDI-TOF spectra of samples before and after DSC study shown in **Figure S11**. Elemental analysis results shown in **Table S1**.

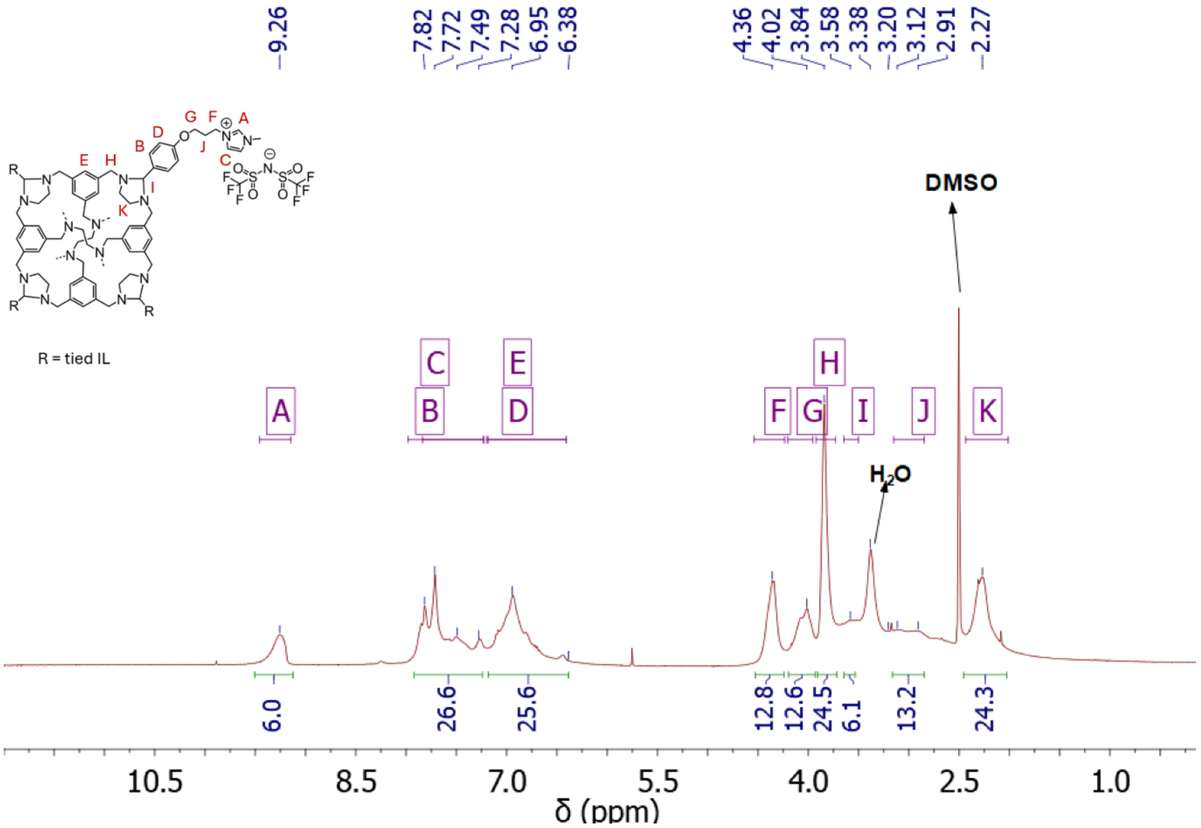

Figure S8: ^{1}H NMR (400 MHz, DMSO-*d*$_6$) of isolated and washed [**RCC1**-IM][NTf$_2$]$_6$.

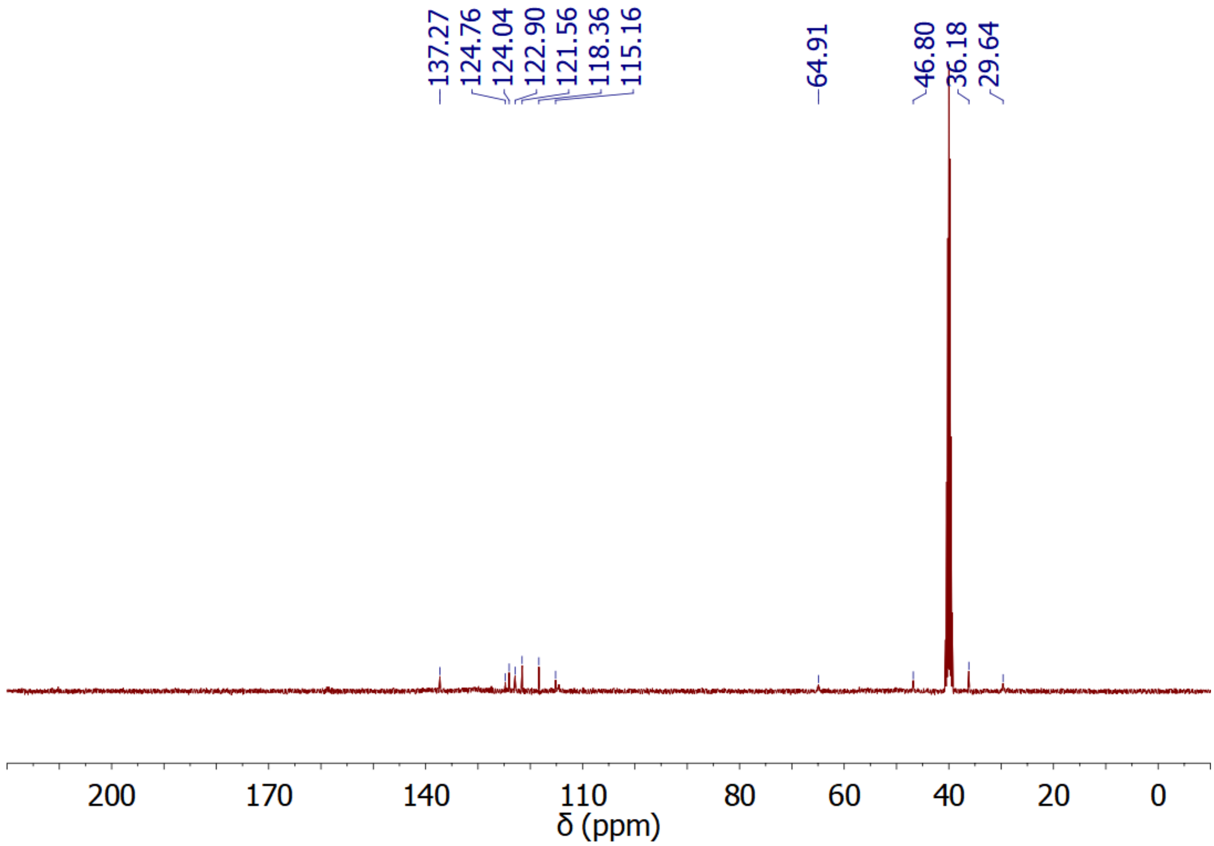

Figure S9: ^{13}C NMR (101 MHz, DMSO-d_6) of isolated and washed [**RCC1**-IM][NTf_2]$_6$.

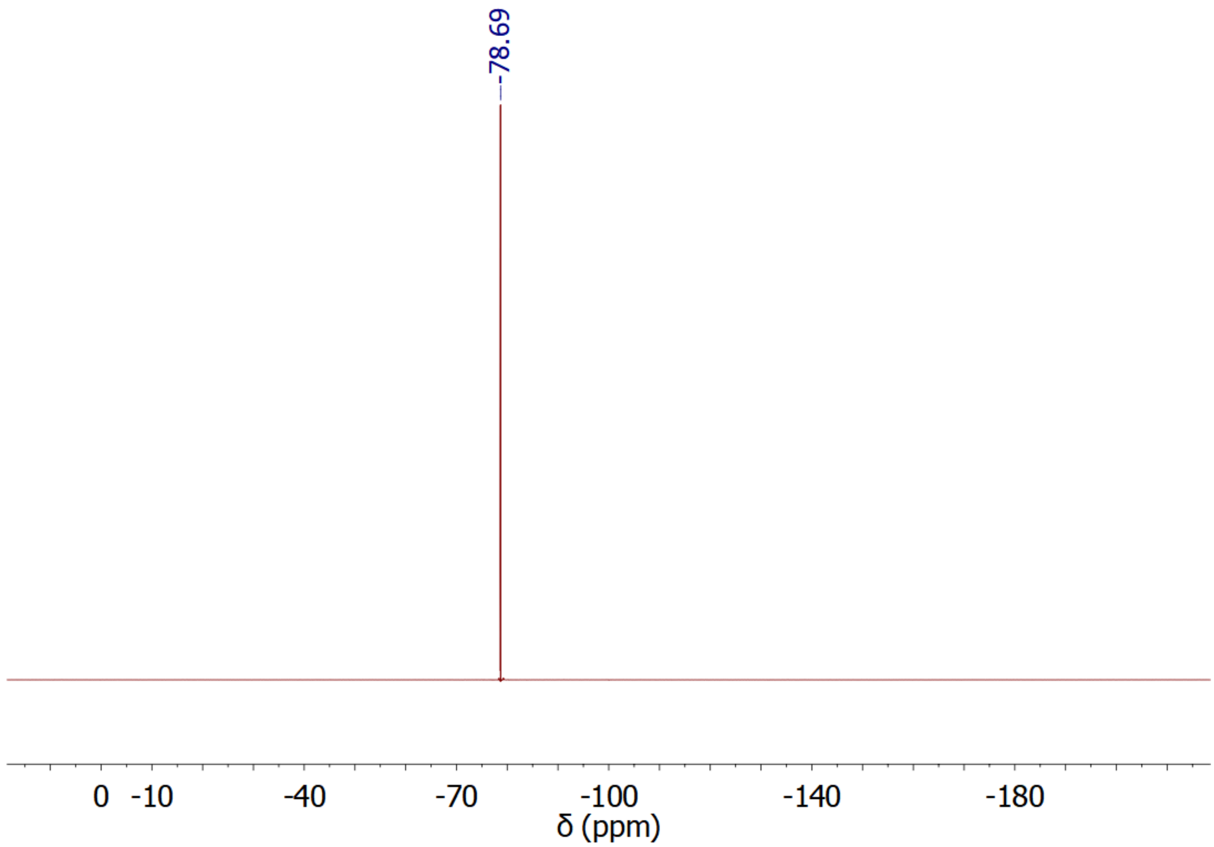

Figure S10: ^{19}F NMR (376 MHz, DMSO-d_6) of isolated and washed [**RCC1**-IM][NTf_2]$_6$.

Table S1: Elemental analysis of [**RCC1**-IM][NTf$_2$]$_6$.

[**RCC1**-IM][NTf$_2$]$_6$ $C_{144}H_{162}F_{36}N_{30}O_{30}S_{12}$	%C	%H	%N	%S
Calculated value	44.79	4.23	10.88	9.96
Analysis 1	44.04	4.19	10.78	-
Analysis 2	43.75	4.13	10.76	-

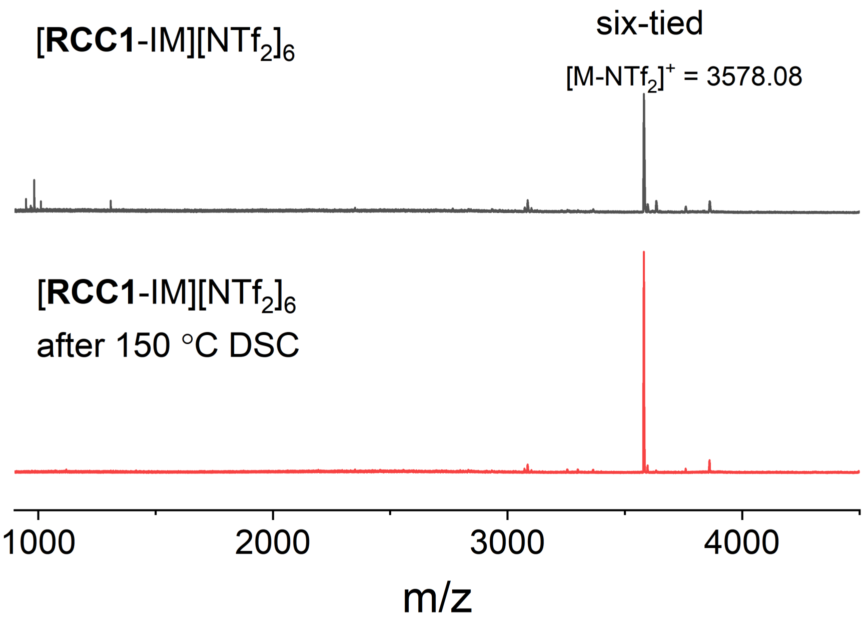

Figure S11: Comparison of MALDI-TOF spectra of [**RCC1**-IM][NTf$_2$]$_6$ before and after DSC study (until 150 °C) in RP mode.

3. Computational Modelling

3.1 Structure and simulation box construction

Here, we describe the structure generation and simulation box construction workflow; the full workflow is detailed and may be reproduced using the corresponding GitHub repository.[3]

The atomistic structure of the [**RCC1**-IM]$^{6+}$ cage was generated using the *stk* software package.[4] Owing to the ionic nature of the cage, the system is manually atom-typed using a combination of the IL.FF[5–8] and OPLS-AA[9] force fields, **Figure S12a**. The individual LAMMPS input files were generated with the *fftool* package.[10]

The atomistic structure of the $[NTf_2]^-$ ion from previously reported studies is used; this structure is described by the IL.FF forcefield, [5–8] an extension of the OPLS-AA forcefield parameterized specifically for ionic liquids, **Figure S12b**. Starting LAMMPS input files were generated with the *fftool* software package.[10]

The simulation box is then constructed, **Figure S12c**. To do this, a single, charge neutral simulation box featuring 1 cage molecule and 6 ions is generated using the *packmol* software package.[11,12] Then this small simulation box is tiled in the *x, y,* and *z* directions using *moltemplate*.[13] This results in the final simulation box consisting of 48 cages and 288 ion molecules.

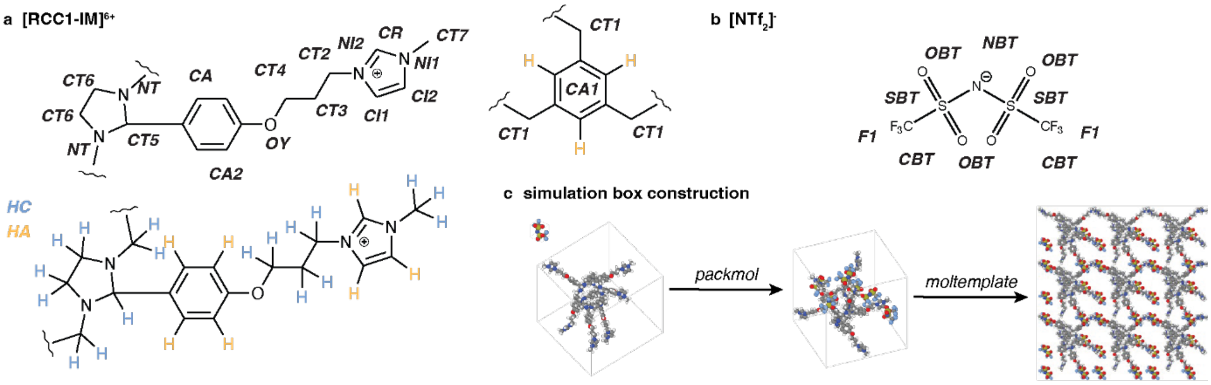

Figure S12: **(a)** [**RCC1**-IM]$^{6+}$ atom types used in simulations. Two types of H atoms are used; HC (blue) and HA (orange). **(b)** $[NTf_2]^-$ atom types used in simulations; these are from the IL.FF presented by Padua *et al.* [5–8] **(c)** Illustration of the simulation box construction; individual cage and ions are packed in a single cell at a cage:solvent ratio of 1:6 to maintain charge neutrality using *packmol.*[11,12] The single cell is tiled using *moltemplate*[13] to generate the full simulation box.

3.2 System equilibration procedure

MD simulations were carried out using LAMMPS; the input files were generated with the *moltemplate* software package[13] in the final simulation box construction step.

To arrive at a reasonable starting geometry, the lattice generated by *moltemplate* (**Figure** S12c) was slowly relaxed. A potential cutoff of 12 Å is used; the partial charges from the IL.FF and OPLS-AA force fields are used to calculate the electrostatic interactions. An NPT ensemble was used with a Nose-

Hoover barostat and thermostat. Simulations were performed at 363 K and 1 atm. Four simulations were performed at increasing time steps: i) 50 fs simulation with a time step of 0.001 fs, ii) 1000 fs simulation with a timestep of 0.01 fs, iii) 5000 fs with a timestep of 0.1 fs, and iv) 0.9 ns with a timestep of 1.0 fs. The simulation box is then thermally annealed using a NVT ensemble. Here, a 1 fs timestep and 100 ps runs, the temperature is stepped from 600 K to 500 K to 400 K to 363 K. Finally, the simulation box was equilibrated for 50 ps using an NPT ensemble and a 1 fs timestep. A production run of 10 ns was then performed at 363 K and 1 atm. **Figure S13** presents the density profile over the 10 ns production run; the density converges to 1.342 $\pm$ 0.005 g/cm^3.

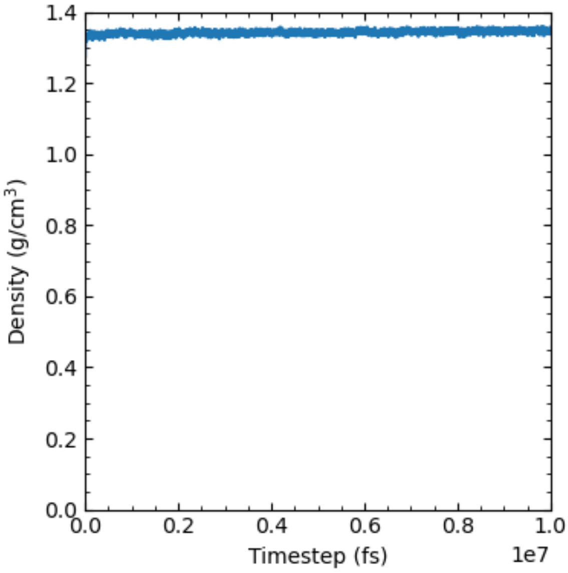

Figure S13. Density profile for the 10 ns production run. The density converges to 1.342 $\pm$ 0.005 g/cm^3

Experimental densities of known NTf_2-based ionic liquids featuring cations containing imidazolium motifs were used, as presented by IL distributor, Ionic Liquid Technologies;[14] specifically, [EMIM][NTf_2], [BMIM][NTf_2] and [HMIM][NTf_2], **Table S2**. The density of [**RCC1**-IM][NTf_2]$_6$ equilibrated to 1.342 +/- 0.006 g/cm^3**. Table S2** details the cationic component of each ionic liquid, the associated density; the density reported for [**RCC1**-IM][NTf_2]$_6$ is calculated from the full liquid simulation. Considering the addition of the cage pores in the PIL, we expect the density to be less than known ionic liquids.

Table S2: Experimental densities for analogous ionic liquids, [EMIM][NTf_2], [BMIM][NTf_2] and [HMIM][NTf_2], as reported by the distributor, Ionic Liquid Technologies.[14] The simulated density for [**RCC1**-IM][NTf_2]$_6$ is also reported.

Ionic Liquid	Cation	Density (g/cm^3)
[EMIM][NTf_2]	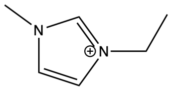	1.52
[BMIM][NTf_2]	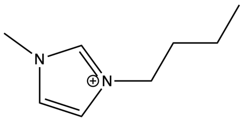	1.44
[HMIM][NTf_2]	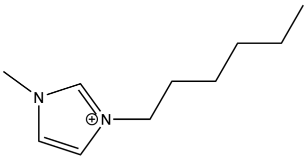	1.37
[**RCC1**-IM][NTf_2]$_6$	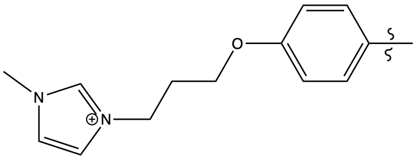	1.34 *(simulated)*

3.3 Radial distribution functions

The radial distribution function (RDF) of several atom pairs is calculated from the 10 ns trajectory, **Figure S14**. We examine the same atom pairs previously reported for [BMIM][NTf_2] simulations.[15] The similarities between the RDFs for these two systems suggests that the liquid structure of the [**RCC1**-IM][NTf_2]$_6$ simulation is reasonable. Further, from the cation-anion RDFs, we observe that the imidazolium rings of the [**RCC1**-IM]$^{6+}$ cage and the imide of the [NTf_2]$^-$ ion are close; this is further supported by the reported trajectory of the [NTf_2]$^-$ counterions in **Figure 4b** of the main text.

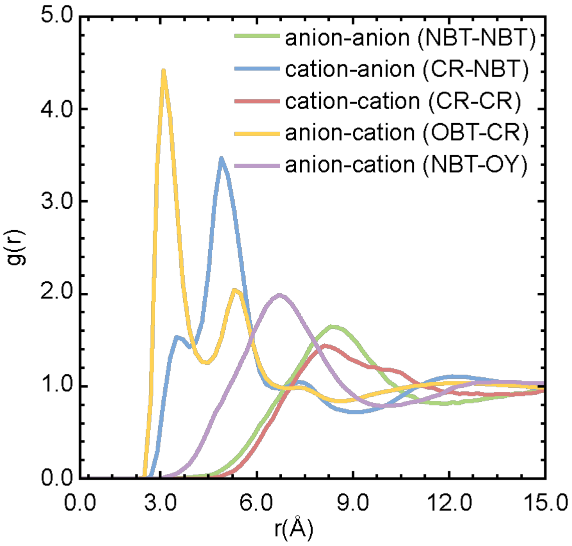

Figure S14 Radial distribution function for the [**RCC1**-IM][NTf_2]$_6$ simulation comparing anionic and cationic component distributions.

3.4 Porosity assessment

Table S3: Criteria used to determine were the [NTf_2]$^-$ ions were over the course of the simulation; distances are measured from the centre of the cage cavity.

Location	Cutoff distance from cage cavity centre (Å)	Average frequency (%)
In bulk solvent	> 20	86.47
Surrounding the cage	5.5 < *[solvent location]* < 20	13.45
In the cage window	3 < *[solvent location]* < 5.5	0.09
Within the cage cavity	< 3	0

4. References

1 S. I. Swamy, J. Bacsa, J. T. A. Jones, K. C. Stylianou, A. Steiner, L. K. Ritchie, T. Hasell, J. A. Gould, A. Laybourn, Y. Z. Khimyak, D. J. Adams, M. J. Rosseinsky and A. I. Cooper, *J. Am. Chem. Soc.*, 2010, **132**, 12773–12775.

2 M. K. Muthayala and A. Kumar, *ACS Comb. Sci.*, 2012, **14**, 5–9.

3 RCC1-IM-NTf_2 https://github.com/austin-mroz/RCC1-IM-NTf2.git.

4 L. Turcani, A. Tarzia, F. T. Szczypiński and K. E. Jelfs, *J. Chem. Phys.*, 2021, DOI: 10.1063/5.0049708.

5 J. N. Canongia Lopes and A. A. H. Pádua, *J. Phys. Chem. B*, 2004, **108**, 16893–16898.

6 J. N. Canongia Lopes, J. Deschamps and A. A. H. Pádua, *J. Phys. Chem. B*, 2004, **108**, 2038–2047.

7 J. N. Canongia Lopes, J. Deschamps and A. A. H. Pádua, *J. Phys. Chem. B*, 2004, **108**, 11250–11250.

8 CL&P force field for ionic liquids https://github.com/paduagroup/clandp.git.

9 L. S. Dodda, I. Cabeza de Vaca, J. Tirado-Rives and W. L. Jorgensen, *Nucleic Acids Res.*, 2017, **45**, W331–W336.

10 FFTool https://github.com/paduagroup/fftool.git.

11 L. Martínez, R. Andrade, E. G. Birgin and J. M. Martínez, *J Comput Chem*, 2009, **30**, 2157–2164.

12 J. M. Martínez and L. Martínez, *J Comput Chem*, 2003, **24**, 819–825.

13 A. I. Jewett, D. Stelter, J. Lambert, S. M. Saladi, O. M. Roscioni, M. Ricci, L. Autin, M. Maritan, S. M. Bashusqeh, T. Keyes, R. T. Dame, J.-E. Shea, G. J. Jensen and D. S. Goodsell, *J. Mol. Bio.*, 2021, DOI: 10.1016/j.jmb.2021.166841.

14 Ionic Liquid Technologies https://iolitec.de/en.

15 M. H. Kowsari, M. Fakhraee, S. Alavi and B. Najafi, *J. Chem. Eng. Data*, 2014, **59**, 2834–2849.
